# Inhibition of Autophagic Flux by Salinomycin Results in Anti-Cancer Effect in Hepatocellular Carcinoma Cells

**DOI:** 10.1371/journal.pone.0095970

**Published:** 2014-05-09

**Authors:** Johannes Klose, Metodi V. Stankov, Moritz Kleine, Wolf Ramackers, Diana Panayotova-Dimitrova, Mark D. Jäger, Jürgen Klempnauer, Michael Winkler, Hüseyin Bektas, Georg M. N. Behrens, Florian W. R. Vondran

**Affiliations:** 1 Department of General, Visceral and Transplantation Surgery, University of Heidelberg, Heidelberg, Germany; 2 Department of General, Visceral and Transplantation Surgery, Hannover Medical School, Hannover, Germany; 3 Department of Clinical Immunology and Rheumatology, Hannover Medical School, Hannover, Germany; 4 Department of Dermatology, Venereology and Allergology, Medical Faculty Mannheim, University Heidelberg, Mannheim, Germany; 5 German Centre for Infection Research (DZIF), partner site Hannover-Braunschweig, Hannover, Germany; University College London, United Kingdom

## Abstract

Salinomycin raised hope to be effective in anti-cancer therapies due to its capability to overcome apoptosis-resistance in several types of cancer cells. Recently, its effectiveness against human hepatocellular carcinoma (HCC) cells both *in vitro* and *in vivo* was demonstrated. However, the mechanism of action remained unclear. Latest studies implicated interference with the degradation pathway of autophagy. This study aimed to determine the impact of Salinomycin on HCC-autophagy and whether primary human hepatocytes (PHH) likewise are affected. Following exposure of HCC cell lines HepG2 and Huh7 to varying concentrations of Salinomycin (0–10 µM), comprehensive analysis of autophagic activity using western-blotting and flow-cytometry was performed. Drug effects were analyzed in the settings of autophagy stimulation by starvation or PP242-treatment and correlated with cell viability, proliferation, apoptosis induction, mitochondrial mass accumulation and reactive oxygen species (ROS) formation. Impact on apoptosis induction and cell function of PHH was analyzed.

Constitutive and stimulated autophagic activities both were effectively suppressed in HCC by Salinomycin. This inhibition was associated with dysfunctional mitochondria accumulation, increased apoptosis and decreased proliferation and cell viability. Effects of Salinomycin were dose and time dependent and could readily be replicated by pharmacological and genetic inhibition of HCC-autophagy alone. Salinomycin exposure to PHH resulted in transient impairment of synthesis function and cell viability without apoptosis induction. In conclusion, our data suggest that Salinomycin suppresses late stages of HCC-autophagy, leading to impaired recycling and accumulation of dysfunctional mitochondria with increased ROS-production all of which are associated with induction of apoptosis.

## Introduction

Salinomycin (Sal) is a polyether antibiotic widely-used as anticoccidial in poultry [1] and dietary supplement in ruminants' and pigs' breed [2], [3] due to its antimicrobial activity. Recently, the potential of Sal as an anti-cancer agent has been elucidated [4]. Gupta et al. demonstrated a more than 100-fold efficiency of Sal compared to Paclitaxel to kill breast cancer stem cells in mice [5]. Later, the efficacy of Sal against tumor cells was reconfirmed in several cancer cell lines from different origin, including solid and non-solid malignancies [6]–[9]. Sal also represents a promising candidate for the treatment of hepatobiliary malignancies as demonstrated for HCC *in* vitro and *in vivo* [10]. We also have shown recently that Sal is feasible to break apoptosis-resistance in human cholangiocarcinoma (CC) cells [11]. Nevertheless, the precise mode of action of Sal as an anti-cancer agent remains unclear. Several mechanisms such as blocking Wingless-type (Wnt)/β-catenin pathway [12] or activation of the conventional caspase driven apoptotic pathways [13] have been proposed. Lately, a new mechanism responsible for the anti-cancer effect of Sal involving drug-mediated alteration of cancer cell autophagic activity was suggested. Autophagy is a recycling pathway for extant proteins, organelles or cellular damage. In tumor cells autophagy is assumed to promote survival. Depending on the experimental system, either inhibition [14] or activation [15]–[17] by Sal of this cellular degradation pathway have been reported.

Thus, aim of this study was to verify the anti-cancer properties of Sal concerning HCC and to elucidate its effect on the pro-survival mechanism of autophagy of these cancer cells. Since pro-apoptotic effects of Sal have been suggested to spare benign cells [9], we further were interested in its impact on primary human hepatocytes (PHH). We have shown that Salinomycin seems to suppress late stages of HCC-autophagy, leading to impaired recycling and accumulation of dysfunctional mitochondria with increased ROS-production and induction of apoptosis. Furthermore, a temporary reduction of cell function in PHH following exposure to Salinomycin was demonstrated.

## Materials and Methods

### Salinomycin

Sal was purchased from Sigma-Aldrich and dissolved in DMSO. Stock solutions were stored at −20°C. Drug concentrations were in the range of similar *in vitro* experiments [10], [11], [14].

### Hepatocyte isolation

Isolation of primary human hepatocytes (PHH) was performed by 2-step collagenase perfusion technique as previously reported [18] (see supporting information). All tissue donors gave written informed consent for experimental use of liver specimen. The protocol was approved by the ethics commission of Hannover Medical School.

### Cell culture

Human HCC cell lines HepG2 (American Type Culture Collection (ATCC), order number HB-8065) and Huh7 [19] were cultured in DMEM (PAA) supplemented with 10% FCS, penicillin (50 U/ml) and streptomycin (50 mg/l) (Invitrogen). Medium was changed every 48 h. For autophagy studies both cell lines were maintained in supplemented ATCC-formulated EMEM as previously described [20]. Established inhibitors and activators of autophagy were used at concentrations previously reported in analogous *in vitro* experiments: 3 MA (0.4–10 mM), LY294002 (0.8–20 µM), vinblastine (0.5–10 µM), nocodazole (0.5–10 µM), PP242 (5 µM), chloroquine (CQ) (5–100 µM) and ammonium chloride (ACH) (1–20 mM) [21]. Physiological induction of autophagy has been performed using HBSS containing 6 mM glucose (starvation medium).

Freshly isolated PHH with a viability of >90% were seeded on collagen-coated 6- and 96-well plates at 1.5 and 0.05×10^6^ viable cells/well, respectively, and cultured using Williams' Medium E as previously described [18]. Following exposure to varying concentrations of Sal (0–10 µM) for 24/48 h, they were further cultured with normal medium for another 5 days. Medium was changed daily. Supernatants and adherent cells were collected for analysis on days 1/3/5.

### Cell viability

PHH and human HCC cells were investigated for *in vitro* cell viability by CellTiter 96AQueous One Solution Cell Proliferation Assay (Promega) as previously described [18]. Furthermore, cell death also was analysed using propidiumiodite (PI) exclusion assay and flow-cytometry. Alternatively apoptosis was analysed using changes in cellular FSC vs. SSC dot plot as previously described [20].

### Proliferation assay

HepG2 and Huh7 cells were cultured at 1×10^3^/well in medium alone or with 1–10 µM Sal in 96-well plates for 24 h. For the last 16 h of culture cells were pulsed with 1 µCi ^3^H-Thymidine and incorporation detected by a β-counter as previously described [11].

### Annexin-V analyses

HepG2 and Huh7 cells were analyzed for apoptosis induction following exposure to 1–10 µM Sal for 24 h applying the Annexin-V apoptosis detection kit (BD Biosciences) according to manufacturer's instructions as previously described [11].

### Constructs and retroviral infection

The plasmids pBABE-puro mCherry-EGFP-LC3B and pBABEpuro GFP-LC3 (N#22418 and 22405), engineered by Jayanta Debnath [21], were obtained from Addgene. Cell transduction and selection were performed as previously described [20], [22]. Genetic inhibition of autophagy was achieved using *ATG7* shRNA-mediated knock down (Santa Cruz). Non-specific shRNA (control) as well as copGFP were applied according to manufacturer's instructions (Santa Cruz). Transduction was performed using lentiviral particles with up to five distinct expression constructs as described elsewhere [20]. Transduction efficiency was quantified by the number of GFP-positive cells and in general exceeded 94% at the end of puromycin selection.

### Analysis of Salinomycin-mediated effects on HCC autophagic activity

Autophagic compartments (pre-autophagosomes, autophagosomes, and autophago-lysosomes) represent intermediate components of a dynamic degradation process and their total amount at a particular time point is determined by the dynamics of their creation and degradation [23]. We conducted studies that measure the autophagic flux according to recent guidelines [23]. Autophagic flux refers to the entire process of autophagy including cargo delivery and subsequent autolysosomal degradation. Measurement of autophagic flux allows the discrimination between induction and late inhibition of autophagosome maturation as both of them are characterized with an increased presence of autophagosomes [23], [24]. Conversion of GFP-LC3-I to GFP-LC3-II was determined by western blotting using anti-LC-3B antibody (Sigma-Aldrich) [23]. Autophagic flux was assessed as accumulation of undegraded GFP-LC3-II after blocking autophagosomal degradation using ACH. Densitometry LC3-II bands was performed using LabImage1D Software (Kapelan Bio-Imaging Solutions) and normalized to the optical density (OD) of actin [24]. Additionally, autophagic flux in HCC cell lines was analyzed by detection of changes in total cellular GFP-LC3 or mCherry-GFP-LC3 signal using flow-cytometry as previously described [20]. Briefly, increased autophagic flux corresponds to a progressive delivery of GFP-LC3 to autolysosomes for degradation and signal disappearance. Furthermore, inhibited autophagic flux translates into reduced GFP-LC3 disappearance and due to the constitutive cellular production is detected as an increased total cellular signal [20]. Analysis was made on LSR-II (BD Biosciences). Autophagic flux also was determined using Cyto-ID Autophagy Detection Kit (Enzo Life Sciences) according to manufacturer's instructions as previously described [25]. This assay is based on a specific dye that selectively stains autophagic compartments. Autophagic flux thus is quantified as accumulation of autophagic compartments in basic or activated conditions (HBSS- or PP242-incubation) after the blockage of autophagolysosomal degradation by CQ or ACH. It is calculated by subtracting the Cyto-ID MFI value of the sample without CQ/ACH from the Cyto-ID MFI value of the sample with CQ/ACH for each condition using the formula: ΔMFI Cyto-ID = MFI Cyto-ID (+CQ/ACH) - MFI Cyto-ID (-CQ/ACH).

### Mitochondrial mass

Mitochondrial mass was determined by flow-cytometry using MitoTracker Green FM (MTR green) staining according to manufacturer's instructions (Molecular Probes) as previously described [26].

### Reactive oxygen species (ROS)

ROS were determined by flow-cytometry applying 5-(and-6)-chloromethyl-2′,7′-dichlorodihydrofluorescein diacetate, acetyl ester (CM-H2DCFDA) staining according to manufacturer's instructions (Invitrogen) as previously described [20].

### Aspartate-aminotransferase leakage and urea formation

As a measure for the degree of cell damage the activity of the aspartate-aminotransferase (AST) was determined for PHH cultures. Furthermore, urea formation as an indicator for cell function was investigated. Both parameters were detected in culture supernatants by standardized enzyme activity assays (Roche Molecular Diagnostics) performed by the central laboratory of Hannover Medical School.

### Albumin synthesis

The synthesis of albumin by PHH was assessed using the Human Albumin ELISA Quantitation Set (Bethyl Laboratories) as previously described [27].

### *In vitro* morphology

The morphology of PHH attached to the collagen-coated plates treated with/without Sal was assessed daily throughout the entire culture period using phase-contrast microscopy.

### Immunofluorescence

PHH were cultured on collagen-coated chamber slides at 5×10^4^ cells/chamber. Following 24 h-incubation with varying concentrations of Sal (0–10 µM) or Fas ligand (FasL) as a positive control (1 µg/ml), staining for apoptosis was performed using the M30 Cytodeath kit (TECOmedical GmbH) according to manufacturer's instructions.

### Statistical analysis

Statistical analysis was performed using SPSS 20.0 and GraphPadPrism 4. The Mann-Whitney-U test, Student´s t-test and ANOVA with Dunnett post-hoc analysis were applied as appropriate. Differences were regarded statistically significant with p<0.05. Results were expressed as mean±SD of at least three independent experiments.

## Results

### Exposure to Salinomycin significantly reduces cell viability and proliferation of HCC cells by induction of apoptosis

**Figure 1:**
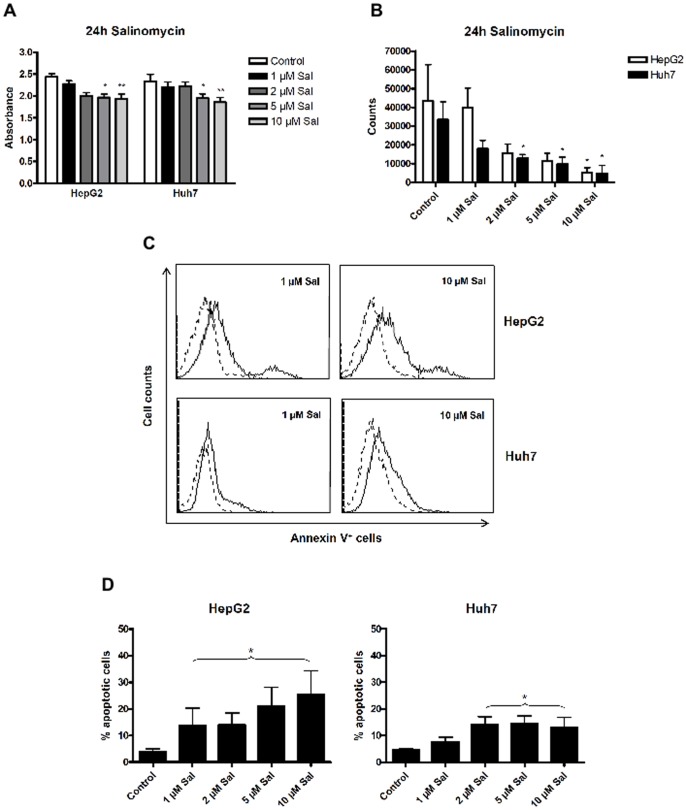
Salinomycin impairs HCC cell survival. HepG2 and Huh7 cells were exposed to increasing concentrations of Salinomycin (1, 2, 5 and 10 µM) for 24 hours. (**A**) Cell viability was assessed by MTS-assay; high concentrations of Salinomycin led to significantly decreased viability of both cell lines (n = 5). (**B**) HCC cells revealed significantly reduced proliferation after exposure to Salinomycin as demonstrated by decreased ^3^H-Thymidine uptake. (**C**) Low concentrations of Salinomycin (left panel, solid line) led to weak increase of apoptotic cells compared to untreated cells (dotted line in overlay). High concentrations markedly induced apoptosis (right panel, solid line). Results are shown as representative scatter-grams of Annexin-V^+^-cells or summarized as mean ± SD of n = 4 independent experiments (**D**). *p<0.05, **p<0.01.

To confirm the anti-cancer effect of Sal on human HCC cells, we investigated cell viability following drug-treatment. For this, HepG2 and Huh7 cells were exposed to increasing concentrations of Sal (1–10 µM) for 24 h followed by analysis using the MTS-assay. Tumor cell viability indeed was reduced significantly at drug concentrations above 2 µM Sal (Fig. 1A). Next, impact on cell proliferation of HCC cells was determined by ^3^H-Thymidine-incorporation after 24 h-incubation with the agent. As demonstrated in Figure 1B, cell proliferation was significantly impaired in both cell lines in a dose-dependent manner. This effect was detectable after exposure to a rather moderate dose of 2 µM Sal. Higher drug concentrations almost completely diminished proliferation of human HCC cells.

We further analysed, whether Sal induced apoptosis in these cell lines. As demonstrated in Figures 1C+D, we observed a dose-dependent increase of Annexin-V^+^ cells following exposure to Sal in HepG2 cells. In Huh7 cells significant apoptosis induction was found at Sal concentrations at and above 2 µM. Induction of apoptosis by Sal was more efficient in HepG2 than in Huh7 cells (25.3% vs. 14.3%).

### Salinomycin inhibits autophagic flux and leads to autophagic substrate accumulation

**Figure 2:**
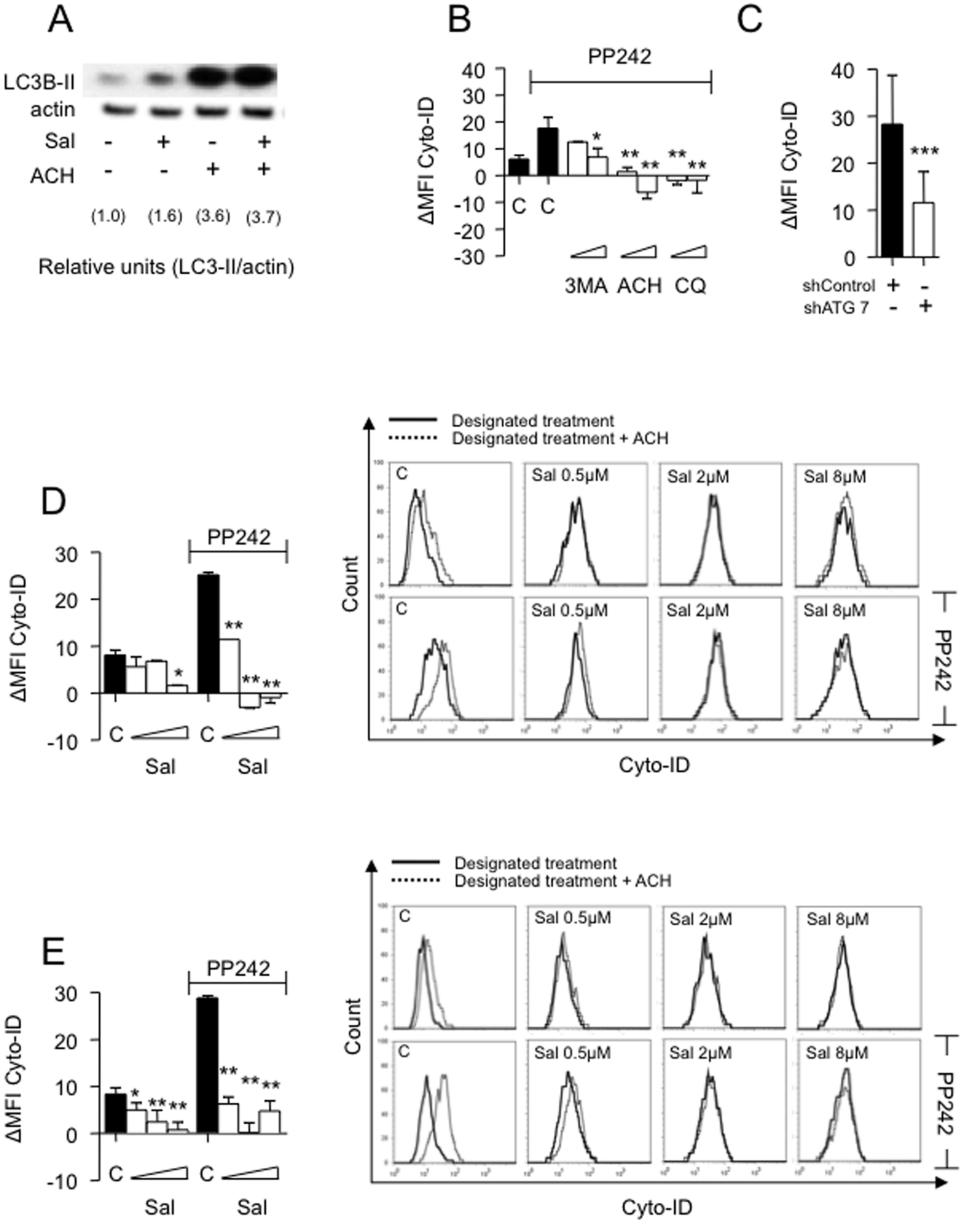
Salinomycin inhibits HepG2 and Huh7 autophagic flux. HepG2 and Huh7 cells were exposed to different concentrations of Salinomycin (0.5, 2 and 8 µM) for different time points and analyzed for activity of autophagy. (**A**) Immunoblot analysis of LC3-I and LC3-II-isoforms (up) with densitometry quantitative analysis (down) in HepG2 cells revealed Sal-induced LC3-II-accumulation due to inhibition of autophagic flux as demonstrated by reduced LC3-II-accumulation after addition of ACH. (**B**) Basic and PP242-activated autophagic flux in Huh7 cells (black bars). Treatment with autophagy inhibitors 3MA (2 and 10 mM), ACH (5 and 20 mM) or CQ (5, 20 µM) for 24 h counteracts PP242 activation of autophagic flux. (**C**) Inhibition of autophagic flux in Huh7 cells after shRNA-mediated knockdown of ATG7. (**D+E**) Decreased accumulation of autophagic compartments after the blockage of autophagolysosomal degradation by ACH indicates reduced autophagic flux in HepG2 (**D**) and Huh7 (**E**) cells treated with Sal for 24 h. Next to the bar graphs representative histograms are depicted. All experiments are presented as mean ± SD of n = 3 independent experiments.*p<0.05; **p<0.01, ***p<0.001.

To assess the effect of Sal on autophagy in HCC cells, cells were cultured in the presence or absence of 10 µM Sal for 15 h with and without ACH for the last 1 h. Semi-quantitative western blot analysis demonstrated Sal-induced increase in LC3-II (Fig. 2A), which argues against a Sal-mediated inhibition of early stages of autophagosomal formation. Instead, this may correspond to either enhanced autophagosome generation due to increased autophagic activity or suppressed autophagosome maturation [23]. To address this question and to assess autophagic flux by western blot we estimated the accumulation of LC3-II band subsequent to ACH-mediated blockage of autophagolysosomal degradation. Accumulation of the LC3-II band was calculated by subtracting the densitometric intensity of the sample without ACH from the sample with ACH for each condition (control 3.6−1.0 = 2.6; Sal 3.7−1.6 = 2.1). Decreased accumulation of the LC3-II band after addition of ACH in Sal-treated cultures (2.6>2.1) suggested drug-mediated suppression of autophagic flux (Fig. 2A). A similar decrease in autophagic flux was detected as a result of vinblastine- and nocodazole-mediated suppression of autophagosome maturation (*data not shown*).

Before we could assess the impact of Sal on HCC autophagic flux, we validated flow-cytometric monitoring of intracellular turnover of autophagic compartments in HCC cells [25], [28]. Autophagy activation by PP242 led to a clear increase in autophagic flux (Fig. 2B). Addition of autophagy inhibitors such as 3-MA, ACH, or CQ resulted in a decrease of autophagic flux. Similar results were obtained using rapamycin or starvation (*data not shown*) and confirmed applicability of the assay in HCC cells. To finally exclude pleiotrophic effects from the pharmacological inhibitors, we verified autophagic flux measurement by Cyto-ID using autophagy-specific shRNA-mediated knockdown of *ATG7* (Fig. 2C).

Next, we wished to evaluate the effect of Sal on HCC's autophagic flux. HepG2 cells were cultured in the presence of Sal (0.5, 2 and 8 µM) for 24 h and compared to untreated cells. Sal noticeably reduced the autophagic flux time- and dose-dependently, even when autophagy was induced by PP242 (Fig. 2D). Similar outcomes were observed using HUH7 cells (Fig. 2E) and alternative activation by rapamycin or starvation (*data not shown*).

**Figure 3:**
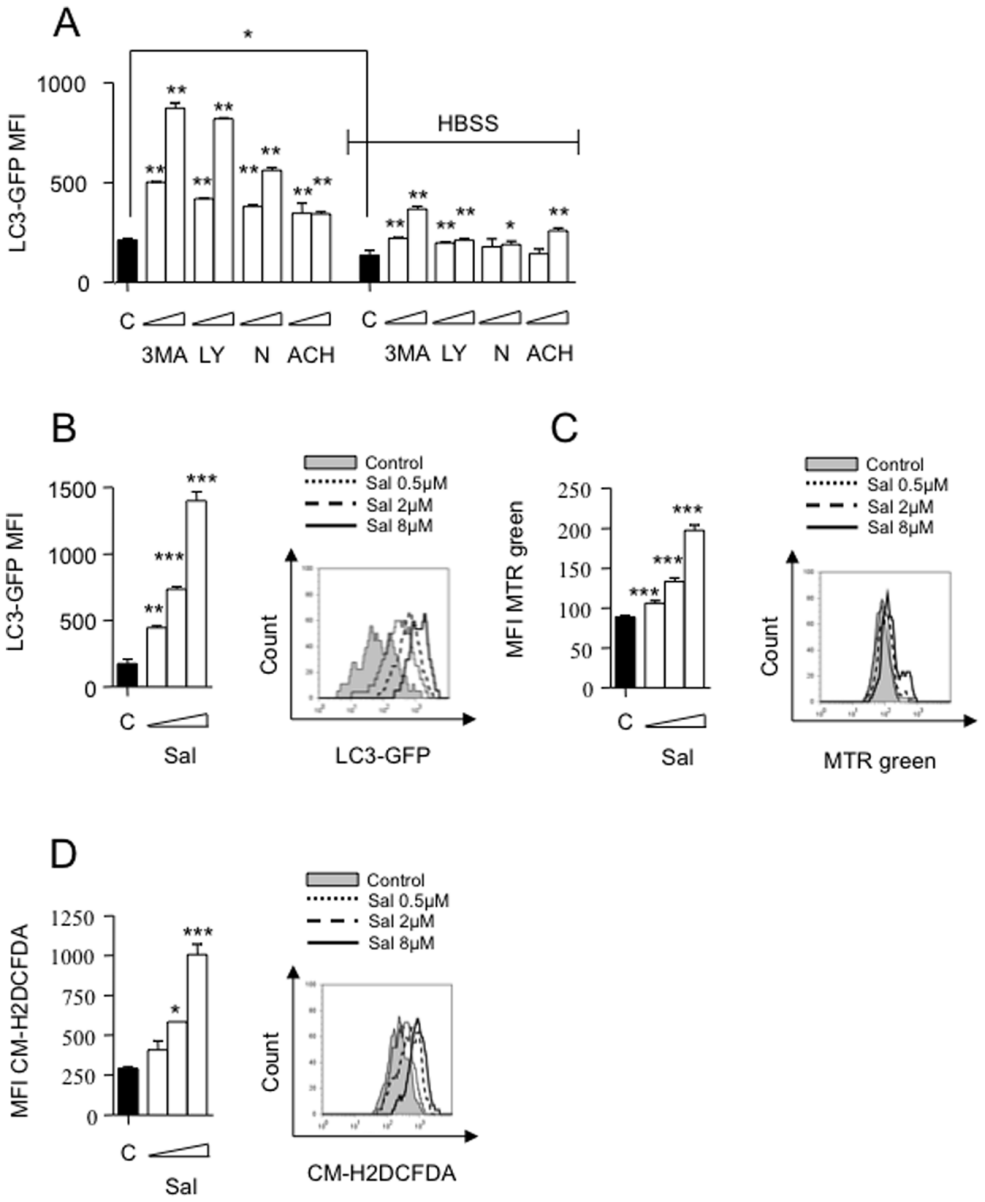
Salinomycin-mediated inhibition of autophagic flux relates to accumulation of dysfunctional mitochondria and increased ROS-production. (**A**) Inhibition of basic and HBSS-activated autophagic flux in HepG2 cells stably expressing LC3-GFP treated with 3 MA (2 and 10 mM), LY (2 and 10 mM), nocodazole (2 and 10 µM) or ACH (0.8 and 4 mM) for 7 h. (**B**) Inhibition of autophagic flux in HepG2 cells treated with Sal (0.5, 2 and 8 µM) for 24 h (left) with representative histograms (right). (**C**) Flow-cytometric analysis of total mitochondrial mass using MitoTracker Green (MTR green) reveals accumulation of dysfunctional mitochondria. (**D**) Evidence of increased ROS-production using CM-H2DCFDA (left) with representative histograms (right). All experiments are presented as mean ± SD of n = 3 independent experiments. *p<0.05, **p<0.01.

Finally, we applied monitoring of intracellular LC3-turnover [22]. For this, we used HepG2 cells stably expressing LC3-GFP. Incubation with 3 MA (2 and 10 mM), LY (2 and 10 mM), nocodazole (2 and 10 µM) or ACH (0.8 and 4 mM) for 7 h lead to accumulation of GFP-LC3 signal, as expected (Fig. 3A). Starvation increased the autophagic flux as detected by decreased LC3-GFP signal in control cells, indicating more degradation (Fig. 3A), an effect which was lost when pharmacological autophagy inhibitors were present (Fig. 3A). Next HepG2 cells constitutively expressing LC3-GFP fusion protein were cultured with and without Sal for 24 h. Sal noticeably inhibited the autophagic flux as detected by accumulation of total cellular LC3-GFP signal (Fig. 3B). Again, Sal's effects on autophagic flux were time- and dose-dependent and readily noticeable at concentrations previously reported to exert anti-cancer effects [10]. Suppression of autophagy has been associated with dysfunctional mitochondria accumulation with increased production of ROS [29], [30], which may trigger apoptosis [29]–[32]. Thus, we analyzed whether Sal treatment was accompanied by accumulation of mitochondrial mass. Indeed, our results confirmed a dose-dependent accumulation of mitochondrial mass (Fig. 3C). Furthermore, accumulated mitochondria displayed signs of dysfunction as demonstrated by an increased presence of ROS production (Fig. 3D). Importantly, pharmacological and genetic inhibition of autophagy in HCC cells completely reiterated Sal effects on dysfunctional mitochondria accumulation, ROS production and cell viability. Based on this we conclude that Sal effects on HCC cells might at least partially be mediated through its ability to suppress autophagic flux (see File S1 and Fig. S1).

### Exposure of PHH to Salinomycin results in transient reduction of cell function and *in vitro* morphology but does not lead to apoptosis

**Figure 4:**
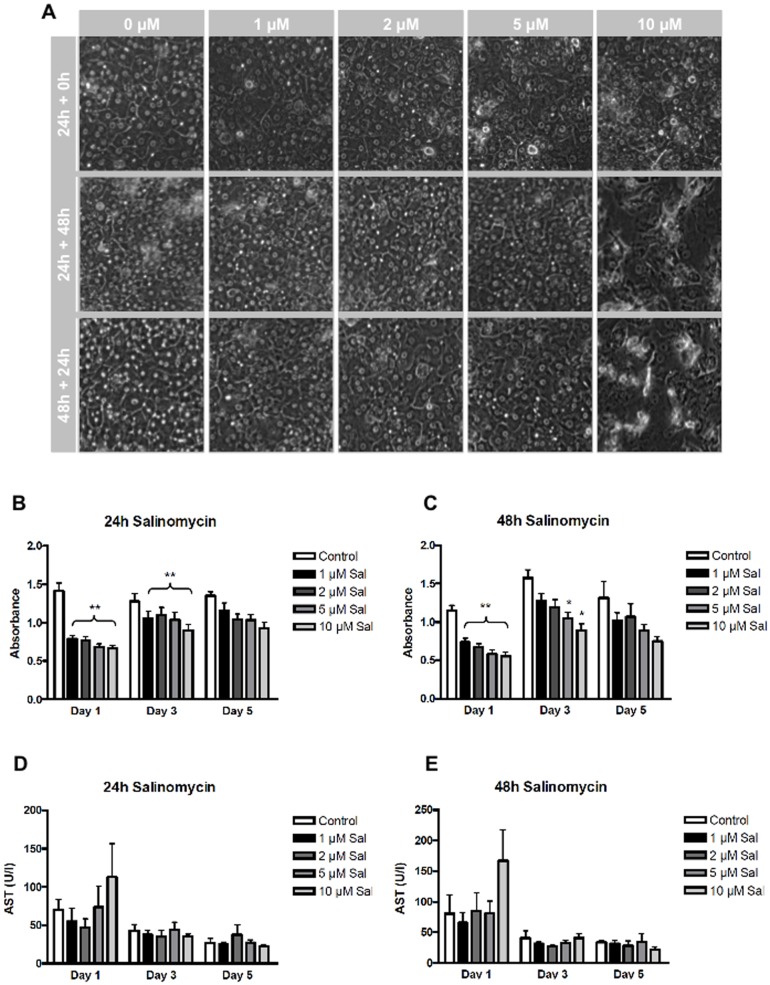
Impact of Salinomycin-treatment on *in vitro* morphology of PHH. After one day of incubation cultured PHH were exposed to increasing concentrations of Salinomycin (1, 2, 5 and 10 µM) at varying time of exposure (24 and 48 hours, respectively). (**A**) Impairment of *in vitro* morphology without subsequent recovery were only detectable after treatment with high concentrations of Salinomycin (10 mM) whereas treatment with up to 5 µM Salinomycin resulted in morphological recovery after lapse of the agent. (**B+C**) Cell viability was assessed by MTS-assay. Salinomycin treatment for 24 (**B**) and 48 (**C**) hours led to significantly impaired cell viability at day 1 and 3. Upon further incubation recovery of the cells was observed as indicated by increasing production of the coloured formazaan-product (n = 6). (**D+E**) Cell damage of PHH as represented by AST release was only detectable immediately after drug exposure (day 1) to 10 µM Salinomycin for 24 (**D**) and 48 (**E**) hours, respectively. This difference did not reach statistical significance (n = 3). Ongoing incubation was accompanied by barely measurable AST release. * p<0.05, ** p<0.005.

Based on reports that Sal exhibits anti-cancer effects against tumor cells but caused little apoptosis in non-tumor cells and the fact that the liver would represent a target organ for the treatment of HCC, we next focused on Sal's effect on primary human hepatocytes. In general, treatment with low concentrations of Sal (1–2 µM) for 24 h did not explicitly alter PHH-appearance (Fig. 4A). Compared to control, hepatocytes appeared slightly affected with irregular cell membranes. Higher concentrations of Sal (5–10 µM) though in part resulted in more obvious alterations: PHH showed signs of integrity destruction and even loss of confluence. Further incubation of these cultures in the absence of Sal led to complete recovery of PHH exposed to low and intermediate doses up to 5 µM. In contrast, after stimulation with 10 µM Sal for 24 h there was no light microscopic detection of effective recovery for most cases. Incubation of PHH for 48 h resulted in similar patterns: treatment with low concentrations of the agent caused rather marginal morphological changes whereas higher doses again could lead to cellular alterations (Fig. 4A).

We next investigated the *in vitro* cell viability of PHH using the MTS-assay. Cell viability was assessed immediately after drug-exposure (day 1) as well as on days 3 and 5 following Sal-treatment. As demonstrated in Figure 4B, 24 h-exposure significantly impaired cell viability at days 1 and 3 in a dose-dependent manner. Lapse of the drug and ongoing incubation led to almost complete recovery of PHH by day 5 (Fig. 4B). Stimulation with Sal for 48 h resulted in a similar dose-dependent decrease of cell viability and subsequent recovery. However, the latter was not as sufficient as observed after 24 h-exposure (Fig. 4C).

Given that incubation of PHH with increasing concentrations of Sal resulted in an interim reduction of cell viability and histomorphologic alterations, we investigated whether other markers of cell damage likewise could be detected. Figures 4D+E demonstrate that comparable leakage of AST was found among all experimental groups. A marked peak of AST-release only was observed at day 1 after exposure to 10 µM Sal but did not reach statistical significance. Ongoing incubation without Sal resulted in moderate basic release of AST in all groups.

**Figure 5:**
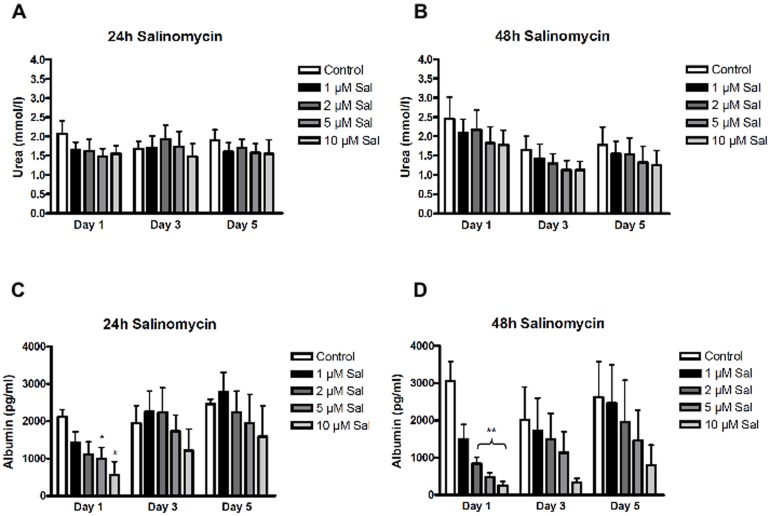
Impact of Salinomycin-treatment on PHH synthesis function. Functionality of PHH after treatment with Salinomycin was analyzed by urea formation and albumin synthesis. (**A**) 24 hours of Salinomycin exposure at varying concentrations was not accompanied by impaired urea formation at all. (**B**) In contrast, treatment for 48 hours resulted in a dose-dependent decrease of urea formation at days 1, 3 and 5 without reaching statistical significance. (**C+D**) Albumin synthesis was markedly impaired at day 1 after stimulation with Salinomycin for 24 and 48 hours, respectively. Further incubation led to continuous recovery of albumin synthesis in the groups with 24 hours of drug exposure. In contrast, treatment for 48 hours resulted only in moderate recovery of albumin synthesis (n = 3). * p<0.05, ** p<0.005.

Next we analyzed if Sal-treatment impairs the function of PHH. For this, urea-formation and albumin-synthesis of drug-exposed PHH were determined. As demonstrated in Figures 5A+B there was no relevant variability in urea-formation after 24 h-stimulation with Sal. In contrast, following 48 h-exposure a dose-dependent decrease of urea-formation was detectable, especially at days 3 and 5, but did not reach statistical significance (Fig. 5A+B). Albumin-synthesis by PHH exposed to Sal for 24 h is characterized by dose-dependent decrease at day 1 (Fig. 5C+D). Lapse of Sal caused a slow recovery of cell functionality with a continuous increase of albumin-production, yet after stimulation with high drug-concentrations. A similar but even more pronounced effect was observed by 48 h-exposure. Again, after lapse of the agent a continuous - though much slower - recovery of PHH was found (Fig. 5C+D).

**Figure 6:**
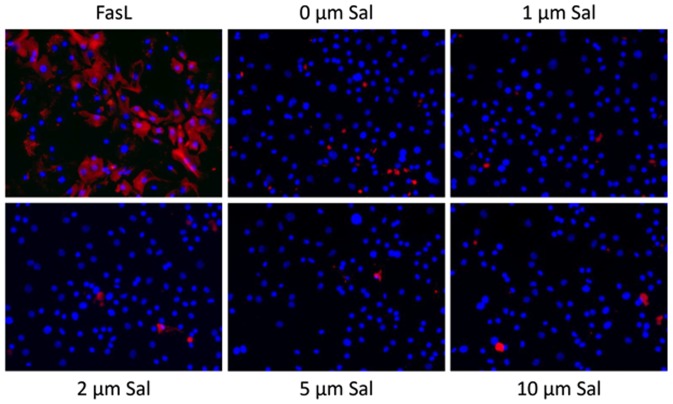
Assessment of apoptosis-induction in PHH by Salinomycin. Cultured PHH were exposed to increasing concentrations of Salinomycin (1, 2, 5 and 10 µM) for 24 hours. Apoptosis was detected by M30 Cytodeath kit. Treatment with Fas Ligand (FasL) served as a positive control. Staining of PHH with DAPI (blue) and M30 Cytodeath kit (red) revealed no evidence for induction of apoptosis in PHH even after treatment with high concentrations of Salinomycin. In contrast, FasL clearly induced apoptosis in PHH.

Finally, we were interested in determining whether the transient impairment of PHH following 24 h-exposure to Sal correlates with apoptosis induction. As shown by Figure 6, exposure to Sal did not provoke apoptosis of these benign cells in contrast to FasL-treatment.

## Discussion

Our data support the notion that Sal exerts a pro-apoptotic effect in HCC cells by inhibition of autophagic flux which leads to accumulation of dysfunctional mitochondria and increased ROS-formation. Given that tumor cells are believed to experience more inherent stress and therefore are predicted to be highly dependent on autophagy for survival [33] our study might help to understand why Sal selectively kills cancer cells while sparing benign cells. This hypothesis is augmented by the observation that exposure of PHH to Sal only results in transient impairment of cell function without substantial damage.

Since HCC is the third leading cause of cancer [34] and the most common primary liver malignancy with limited treatment-options particularly at advanced stage of disease [34], [35], innovative therapeutic approaches are essential. The potential of Sal for the treatment of HCC was firstly suggested by the work of Wang et al. who demonstrated inhibition of proliferation and induction of apoptosis in HCC *in vitro* and *in vivo* after drug-exposure [10]. To dissect the precise mode of action of Sal we focused on the molecular mechanism of Sal in HCC. Using HepG2 and Huh7 cells and comprehensive quantitative analyses we were able to demonstrate that Sal inhibits autophagic flux in human HCC cells. The following findings led us to the conclusion that Sal-mediated inhibition of autophagic flux might be responsible for apoptosis in HCC cells. First, we demonstrated a dose-dependent accumulation of dysfunctional mitochondria with increased ROS-production. Elimination of dysfunctional mitochondria and thus reduction of pro-apoptotic signals such as cytochrome *c* release are considered to be an integral part of the pro-survival mechanism of autophagy [36]. Of note, autophagy has been repeatedly shown to be crucial for the removal of dysfunctional mitochondria [31], [37]. Detectable alteration of mitochondrial mass and ROS appeared only after substantial inhibition of autophagic flux and was followed by a massive induction of apoptosis and cell death. Second, under our experimental conditions pharmacological and genetic inhibition of autophagy entirely reiterated these events. Even if one considers that almost all of the pharmacological inhibitors present pleiotropic effects on multiple pathways [23], the fact that genetic inhibition resulted in analogous alteration argues for the possibility that our observations are at least partially mediated by the suppression of autophagic flux. The impact of Sal on autophagy in cancer cells is currently discussed controversially. Jangamreddy et al. proposed that induction of autophagy by Sal in prostate and breast cancer cells is associated with cell protective properties [15]. Similar to our findings the group also observed mitochondrial disintegration and increased mitochondrial mass. The toxic effects of Sal were explained by mitochondrial hyperpolarization and ATP-depletion. Another study reports that Sal-induced autophagy by endoplasmatic reticulum stress response in lung cancer cells [16]. In combination with autophagy inhibitor-treated cells an increased amount of apoptotic cells was detected. While those studies conclude that Sal induces autophagy in cancer cell lines, Yue et al. have demonstrated that inhibition of autophagic flux in breast cancer stem-like/progenitor cells induces apoptosis [14]. We conclude on these reports and our findings that the impact of Sal on autophagy might vary between tumor cells of different origin.

Next, we focused on toxicity in primary human hepatocytes. The importance of this issue is highlighted by the fact that letal intoxication of Sal has been reported in humans and animals [38]-[40]. It has been demonstrated that non-malignant cells are excluded from the effects of Sal [9], [41]. Our findings indicate that Sal seems to exert some toxic effects on PHH (increased AST-release, decrease of albumin and urea production) at higher doses (10 µM) but at lower concentrations is well tolerated. Concerning the capacity to recover following Sal-exposure, a certain degree of variation between donor to donor in these primary cells was observed. Differences in autophagic potential of these benign cells and/or general cell quality might account for this effect. It is remarkable that even stimulation for 48 h with high drug-concentrations could result in complete recovery of PHH. Furthermore, Sal did not induce apoptosis in PHH. Even Wang et al. have shown in their *in vivo* studies that Sal does not induce apoptosis in livers of mice [10]. Although the initial viability of PHH was decreased after treatment substantial recovery for most human hepatocytes following Sal-treatment could be demonstrated.

In conclusion, our data confirm the potent anti-cancer effect of Sal as demonstrated by marked impairment of cell viability and proliferation of treated HCC cell lines. Furthermore, we provide for the first time a mode of action of Sal in HCC cells – suppression of late stages of autophagy with subsequent induction of apoptosis. Inhibition of autophagic flux thus represents a potential explanation for Sal's selectivity for cancer cells. In addition, it was shown that Sal does not exert severe hepatotoxic effects per se but at least at higher concentrations causes transient impairment of hepatocyte function. Further studies now are required to confirm the mechanism of action of Sal in other types of cancer including development of innovative animal models to determine efficacy and potential risks of this drug *in vivo*.

## Supporting Information

Figure S1 Pharmacological and genetic inhibition of autophagy recapitulates the effects of Salinomycin on HCC. Flow-cytometric analyses of HepG2 for (**A**) apoptosis, (**C**) total mitochondrial mass using MTR green and (**E**) ROS-production using CM-H_2_DCFDA following treatment with 3 MA (0.4, 2 and 10 mM), LY (0.4, 2 and 10 mM), nocodazole (0.4, 2 and 10 µM), vinblastine (0.4, 2 and 10 µM), ACH (0.8, 4 and 20 mM) or CQ (4, 20 and 100 µM) for 48 h. For genetic knock-down, Huh7 cells were transduced with lentiviral particles expressing shATG7 or non-specific shRNA control. Flow-cytometric analyses of (**B**) apoptosis, (**D**) total mitochondrial mass using MTR green and (**F**) ROS-production using CM-H_2_DCFDA 72 h after puromycin selection. Data is presented as mean±SD and representative for at least three independent experiments with two to four replicates. *p<0.05; **p<0.01; ***p<0.001.
(TIFF)

File S1 A brief description of hepatocyte isolation and culture is presented. Additionally, by pharmacological and genetic inhibition of autophagy in HepG2 and Huh7 cells the effects of Salinomycin on HCC can be recapitulated.
(DOCX)
