# Supplementary material for: Inhibition of Autophagic Flux by Salinomycin Results in Anti-Cancer Effect in Hepatocellular Carcinoma Cells
Source: PLoS One. 2014 May 9;9(5):e95970. doi: 10.1371/journal.pone.0095970 (PMC4015957; doi:10.1371/journal.pone.0095970)
Supplement: File S1 — A brief description of hepatocyte isolation and culture is presented. Additionally, by pharmacological and genetic inhibition of autophagy in HepG2 and Huh7 cells the effects of Salinomycin on HCC can be recapitulated. (DOCX) [file pone.0095970.s002.docx]

**Supporting information**

*Hepatocyte isolation*

In brief, liver specimen obtained after partial hepatectomy were cannulated under sterile conditions and flushed once with washing buffer containing 2.5 mM EGTA. This was followed by recirculating perfusion with digestion buffer containing 0.05 % collagenase (Roche P, Mannheim, Germany). The resulting cell pellet after mechanical disruption of the tissue was washed twice using PBS and resuspended in supplemented William´s medium E (all Biochrom AG, Berlin, Germany) (1 µM insulin, 1 µM dexamethason/fortecortin, 100 U/ml penicillin, 100 µg/ml streptomycin, 1mM sodium pyruvate, 15 mM HEPES buffer, 4 mM L-glutamine and 5 % FCS). Cell number and viability were determined by the Trypan blue exclusion test.

*Pharmacological and genetic inhibition of autophagy recapitulates the effects of Salinomycin on HCC*

Taking into account that our previous experiments suggested Sal-induced inhibition of autophagic flux, we aimed to address the issue whether autophagy inhibition represents a critical step contributing to Sal-toxicity in HCC. To this end we incubated Huh7 and HepG2 cells with and without 3MA (0.4, 2 and 10 mM), LY (0.4, 2 and 10 mM), nocodazole (0.4, 2 and 10 µM), vinblastine (0.4, 2 and 10 µM), ACH (0.8, 4 and 20 mM) or CQ (4, 20 and 100 µM) for 48 h. We used these pharmacological inhibitors to evaluate the effects of autophagy inhibition on cell proliferation, cell viability, mitochondrial accumulation and ROS-formation in HCC. Our results verified that autophagy inhibition at various stages suppresses HepG2-growth as demonstrated by decreased cell numbers (*data not shown*) and induced apoptosis and cell death (Supporting Fig. S1A and *data not shown*). In line with this, we were able to show that inhibition of autophagic flux (Fig. 2B and Fig. 3A) resulted in an accumulation of dysfunctional mitochondria (Supporting Fig. S1C) with increased ROS-production (Supporting Fig. S1E). Similar results were obtained using Huh7 cells (*data not shown*).

In order to check for the potential pleiotropic effects of pharmacological inhibitors we took advantage of shRNA-mediated knockdown of *ATG7*, which is essential for the process of autophagy. Genetic inhibition of autophagy in Huh7 cells confirmed reduced cell numbers (*data not shown*), increased apoptosis (Supporting Fig. S1B), accumulation of dysfunctional mitochondria (Supporting Fig. S1D) and higher ROS-production (Supporting Fig. S1F). Similar results were obtained using HepG2 cells (*data not shown*).

Therefore, genetic and pharmacologic inhibition of autophagic flux translates into accumulation of dysfunctional mitochondria with higher ROS-formation and triggers apoptosis and growth arrest in HepG2 and Huh7 cell lines, recapitulating the effects of Sal.

**Supporting figure legend**

Figure S1: *Pharmacological and genetic inhibition of autophagy recapitulates the effects of Salinomycin on HCC.*

Flow-cytometric analyses of HepG2 for **(A)** apoptosis, **(C)** total mitochondrial mass using MTR green and **(E)** ROS-production using CM-H_2_DCFDA following treatment with 3MA (0.4, 2 and 10 mM), LY (0.4, 2 and 10 mM), nocodazole (0.4, 2 and 10 µM), vinblastine (0.4, 2 and 10 µM), ACH (0.8, 4 and 20 mM) or CQ (4, 20 and 100 µM) for 48 h. For genetic knock-down, Huh7 cells were transduced with lentiviral particles expressing shATG7 or non-specific shRNA control. Flow-cytometric analyses of **(B)** apoptosis, **(D)** total mitochondrial mass using MTR green and **(F)** ROS-production using CM-H_2_DCFDA 72 h after puromycin selection. Data is presented as mean±SD and representative for at least three independent experiments with two to four replicates. *p <0.05; **p <0.01; ***p <0.001.
